# Symbiotic relationship between *Polyporus umbellatus* and *Armillaria gallica* shapes rhizosphere bacterial community structure and promotes fungal growth

**DOI:** 10.3389/fmicb.2025.1658060

**Published:** 2025-09-11

**Authors:** Lingfeng Zhou, Liu Liu, Weiwei Gao, Bing Li, Shunxing Guo

**Affiliations:** ^1^Institute of Medicinal Plant Development, Chinese Academy of Medical Sciences and Peking Union Medical College, Beijing, China; ^2^State Key Laboratory for Quality Ensurance and Sustainable Use of Dao-di Herbs, Beijing, China

**Keywords:** *Polyporus umbellatus*, *Armillaria gallica*, symbiosis, rhizosphere bacterial communities, *Rhodococcus* sp.

## Abstract

**Aims:**

*Polyporus umbellatus* sclerotium, known for its diuretic properties, relies on a symbiotic association with *Armillaria* for its growth and quality development. However, the impact of soil microorganisms on this symbiosis remains uncertain and warrants investigation. The primary objective of this research is to characterize the microorganisms capable of enhancing the symbiotic interaction between *Armillaria gallica* and *Polyporus umbellatus* sclerotia in the rhizosphere soil.

**Methods:**

Symbiotic cultivation experiments were conducted in woodland habitats with four groups: symbiotic group (Z0), control group (Z1), *A. gallica*-only group (Z2), and *P. umbellatus*-only group (Z3). Rhizosphere soil community profiling analysis was conducted using high-throughput sequencing of the bacterial 16S rRNA gene. Subsequently, bacterial strains were isolated, purified, and back-inoculated with *A. gallica* to assess their effects on this symbiotic relationship.

**Results:**

A total of 10,009 operational taxonomic units (OTUs) were identified, with the symbiotic group (Z0) showing higher bacterial richness and diversity (ACE, Chao1, Shannon indices) compared to Z2 and Z3. Dominant phyla such as Proteobacteria, Acidobacteriota, and Bacteroidota were notably more abundant in Z0. Notably, *Rhodococcus* sp. Z2-1 significantly promoted *A. gallica* rhizomorph growth (diameter increased by 112.2%, branches by 160.9%) and symbiosis establishment (100% contact rate in inoculated pots vs. 0–22.2% in controls).

**Conclusion:**

The symbiotic relationship between *P. umbellatus* and *A. gallica* shapes rhizosphere bacterial communities, with specific bacteria like *Rhodococcus* sp. enhancing fungal growth and symbiotic efficiency. This study presents the potential for developing a bio-bacterial fertilizer for cultivation of medicinal material.

## Introduction

*Polyporus umbellatus* (Pers.) Fries, a valuable medicinal fungus utilized in traditional medicine for its therapeutic attributes (Gao et al., 2025), depends significantly on its symbiotic relationship with *Armillaria* spp., which play a crucial role in influencing the growth rate, yield, and bioactive compound accumulation in *P. umbellatus* (Liu et al., 2025). Despite its medicinal significance, the extended natural growth cycle and inconsistent yield of *P. umbellatus* limit its widespread application. Therefore, a comprehensive understanding of the symbiotic mechanisms between *P. umbellatus* and *A. gallica* is essential for improving the efficiency and quality of *P. umbellatus* cultivation.

The rhizosphere microbiome, comprising the diverse microbial communities in the soil surrounding plant roots and fungal mycelia, plays a crucial role in the growth and development of both plants and fungi (Xun et al., 2024). These microbial communities engage in interactions with their hosts, influencing processes such as nutrient uptake, growth promotion, and disease resistance (Elnahal et al., 2022). Recent advancements in microbiomics have provided new methodologies for investigating the structure and functionality of rhizosphere microbial communities. Research indicates that the diversity and functions of rhizosphere microbiota are crucial for shaping symbiotic relationships (Wang et al., 2024). Nevertheless, research on the rhizosphere microbiome associated with *P. umbellatus* remains limited, especially regarding the dynamic changes and functional roles of microbial communities in the symbiotic system between *P. umbellatus* and *A. gallica*.

In the symbiotic relationship between *P. umbellatus* and *A. gallica*, the rhizosphere microbiome potentially influences the initiation and maintenance of this symbiosis through various mechanisms. A diverse microbial community can provide a plentiful nutrient source, thereby promoting the growth and development of both *P. umbellatus* and *A. gallica*. Moreover, specific microbial taxa may impact the growth of *P. umbellatus* and *A. gallica* directly or indirectly by generating plant growth-promoting compounds, facilitating phosphate solubilization, nitrogen fixation, and other functions. Additionally, the interactions within the microbial community can impact the disease resistance and adaptability of *P. umbellatus* and *A. gallica*. Nonetheless, the specific composition, functions, and mechanisms of the rhizosphere microbiome in this symbiotic system remain unclear.

This study aims to investigate the structure and diversity of bacterial communities in the rhizosphere soil of *P. umbellatus* and explore their roles in the symbiotic association of *P. umbellatus* and *A. gallica*. Utilizing high-throughput sequencing, we analyzed the bacterial communities across various experimental groups, including the symbiotic group, *P. umbellatus*-only group, *A. gallica*-only group, and control group. Furthermore, we isolated and identified specific bacterial strains and conducted co-culture experiments to assess their effects on the growth of *P. umbellatus* and *A. gallica*. The results of this study are anticipated to provide valuable theoretical insights into the microbial mechanisms underpinning the symbiotic relationship between *P. umbellatus* and *A. gallica*, offering scientific guidance for improving *P. umbellatus* cultivation and quality.

## Materials and methods

### The co-cultivation of *Polyporus umbellatus* sclerotia and *Armillaria gallica*

*P. umbellatus* sclerotia were cultivated in woodland habitats in Liuba County, Shaanxi Province, China (33 °30′ N, 106 °59′E). The experimental design consisted of four groups: the experimental group (Z0), the control group (devoid of both *P. umbellatus* and *A. gallica* in comparison to the experimental group) (Z1), the *A. gallica*-only group (containing *A. gallica* but lacked *P. umbellatus* compared to the experimental group) (Z2), and the *P. umbellatus*-only group (including *P. umbellatus* but lacking *A. gallica* compared to the experimental group) (Z3). A total of 30 holes were excavated in each group, measuring 50 cm in length, 30 cm in width, and 15 cm in depth. In each hole of the experimental group, one wooden stick (approximately 15 cm in diameter and 30 cm in length) was planted, along with 150 g of immature *P. umbellatus* sclerotia and 1,500 g of *A. gallica*-infected smaller wooden sticks (approximately 3 cm in diameter and 8 cm in length). Following a six-month cultivation period, rhizosphere soil samples of *P. umbellatus* were collected monthly, with five pits sampled during each collection. This approach ensured the acquisition of rhizosphere soil samples throughout the symbiotic phase between *P. umbellatus* and *A. gallica*.

### Collection of soil samples

The experimental design and sample collection were adapted from the methods described by Zhao et al. (2024), with specific modifications to suit the current study. All soil subsamples collected during the same sampling period and treatment were homogenized and amalgamated into composite samples, which were subsequently transported to the laboratory in a foam box with dry ice. Upon arrival, the samples were stored at −80 °C for DNA extraction and subsequent high-throughput sequencing analysis. In the study design, Z0 represents soil from the experimental group, Z1 denotes soil from the control group, Z2 refers to soil from the *A. gallica*-only group, and Z3 indicates soil from the *P. umbellatus*-only group.

### DNA extraction and 16S rRNA gene sequencing

Genomic DNA from soil samples was extracted using the cetyltrimethylammonium bromide (CTAB) method. Four soil samples were collected from each habitat. High-throughput sequencing of bacteria was performed using the 16S rRNA gene V3-V4 hypervariable region primers 338F (5′-ACTCCTACGGGAGGCAGCAG-3′) and 806R (5′-GGACTACHVGGGTWTCTAAT-3′). The PCR system and conditions were consistent with prior work (Bo et al., 2024). Following PCR, product quantification, purification, library preparation, and 16S rRNA amplicon sequencing were performed by Majorbio Bio-pharm Technology Co., Ltd. (Shanghai, China). Sequencing was performed on the Illumina NextSeq 2000 Sequencing Platform, generating 300 bp paired-end reads for 16S rRNA amplicon sequencing. A total of 1,072,758 raw reads were obtained through sequencing, and after quality filtering, 1,040,974 valid reads were obtained. The average sequencing depth of each sample was 52,049 reads/sample (range: 44,154–57,634 reads), ensuring that the sequencing depth of each sample was sufficient to cover community diversity.

### Diversity of bacterial communities

The obtained data were processed using the QIIME software platform. The operational taxonomic units (OTUs) representing bacterial species in the 16S OTU table were identified. OTU clustering was performed with a 97% sequence similarity threshold. Species annotation analysis of the OTU sequences was conducted using the QIIME software and the SSU rRNA database SILVA138. Additional analyses of bacterial community diversity were carried out using I-Sanger Bioinformatics Cloud Platform (version 3.0) at https://cloud.majorbio.com. The bacterial co-occurrence interaction network was explored using Networkx (version1.11) to identify significant correlations between bacterial taxa (Maurice et al., 2024).

### Isolation and identification of microbial communities associated with *Polyporus umbellatus*

A total of 10 g soil on the surface of *P. umbellatus* sclerotia was added in 50 mL of ddH_2_O with orbital shaker (120 rpm and 30 min). The resulting solutions were then diluted 10^4^-fold. Subsequently, 100 μL of the diluted solution was spread onto various media, including nutrient agar, Luria–Bertani medium (LB), potato dextrose agar (PDA) medium and tryptone soya agar. Incubation of plates occurred at 25 °C for 2–7 days (Gao et al., 2024). Sequencing of the bacterial 16S rRNA gene and fungal ITS gene followed established protocols (Ying et al., 2012; Handique et al., 2024). PCR products were sequenced by Taihe Biotechnology Co., Ltd. (Beijing, China). Sequence identification was performed using EzBioCloud and NCBI BLAST databases (Chalita et al., 2024).

### Co-culture experiments of isolated bacteria and *Armillaria gallica*

The impact of bacteria on growth rate and branching number of the rhizomorph of *A. gallica* was assessed (Liu et al., 2022). The bacteria were cultured in LB medium overnight at 28 °C with shaking at 180 rpm until reaching an optical density (OD600) of 0.5. Rhizomorph tips of *A. gallica* (approximately 2 mm in length) were placed at the center of PDA plates, with bacteria spread 2 cm away from both sides of the rhizomorphs. Control plates lacking bacteria were included. All plates were incubated in darkness at 25 °C for 14 days. The experiment was conducted with three replicates (*n* = 3), and measurements were taken for the diameter and branching number (branch length exceeding 1 mm) of the *A. gallica* rhizomorphs.

### Pot experiment to assess the effect of *Rhodococcus* sp. strain Z2-1 on symbiosis establishment between *Polyporus umbellatus* and *Armillaria gallica*

*Rhodococcus* sp. strain Z2-1, previously isolated from the rhizosphere soil of *P. umbellatus*, was used to assess its impact on the symbiotic relationship establishment between *P. umbellatus* and *A. gallica*. A pot experiment was conducted with two treatments: a control group (normal cultivation) and an experimental group (inoculated with Z2-1). Each treatment consisted of five replicates planted in glass containers (351 × 211 × 250 mm) filled with sandy soil sterilized at 121 °C for 40 min. Each container was planted with a wooden stick (8 cm in diameter, 25 cm in length), 100 g of immature *P. umbellatus* sclerotia, and 1,000 g of smaller wooden sticks (5 cm in length, 2 cm in diameter) colonized by *A. gallica*. *Rhodococcus* sp. strain Z2-1 was cultured in 200 mL of Luria-Bertani (LB) medium at 28 °C with shaking at 180 rpm for 24 h. Bacterial cells were harvested by centrifugation at 4,000 rpm for 10 min, washed twice with saline, and resuspended in sterile distilled water to a final concentration of 1 × 10^8^ CFU/mL. The experimental group received inoculation with this bacterial suspension, while the control group was treated with an equivalent volume of sterile distilled water. *P. umbellatus* was cultivated in darkness at room temperature for 6 months. Subsequently, the symbiotic rate between *P. umbellatus* and *A. gallica* was assessed to determine the symbiotic-promoting ability of *Rhodococcus* sp. strain Z2-1.

### Statistical analysis

Microbial community analyses were conducted using the R statistical program, version 3.3.0. Student’s *t*-test was employed to compare the differences between the control and treatment groups. Results are presented as means ± SEM; *p < 0.05; **p < 0.01; ***p < 0.001.

## Result

### Microbial communities in the soil of the *Polyporus umbellatus* habitat

**Figure 1:**
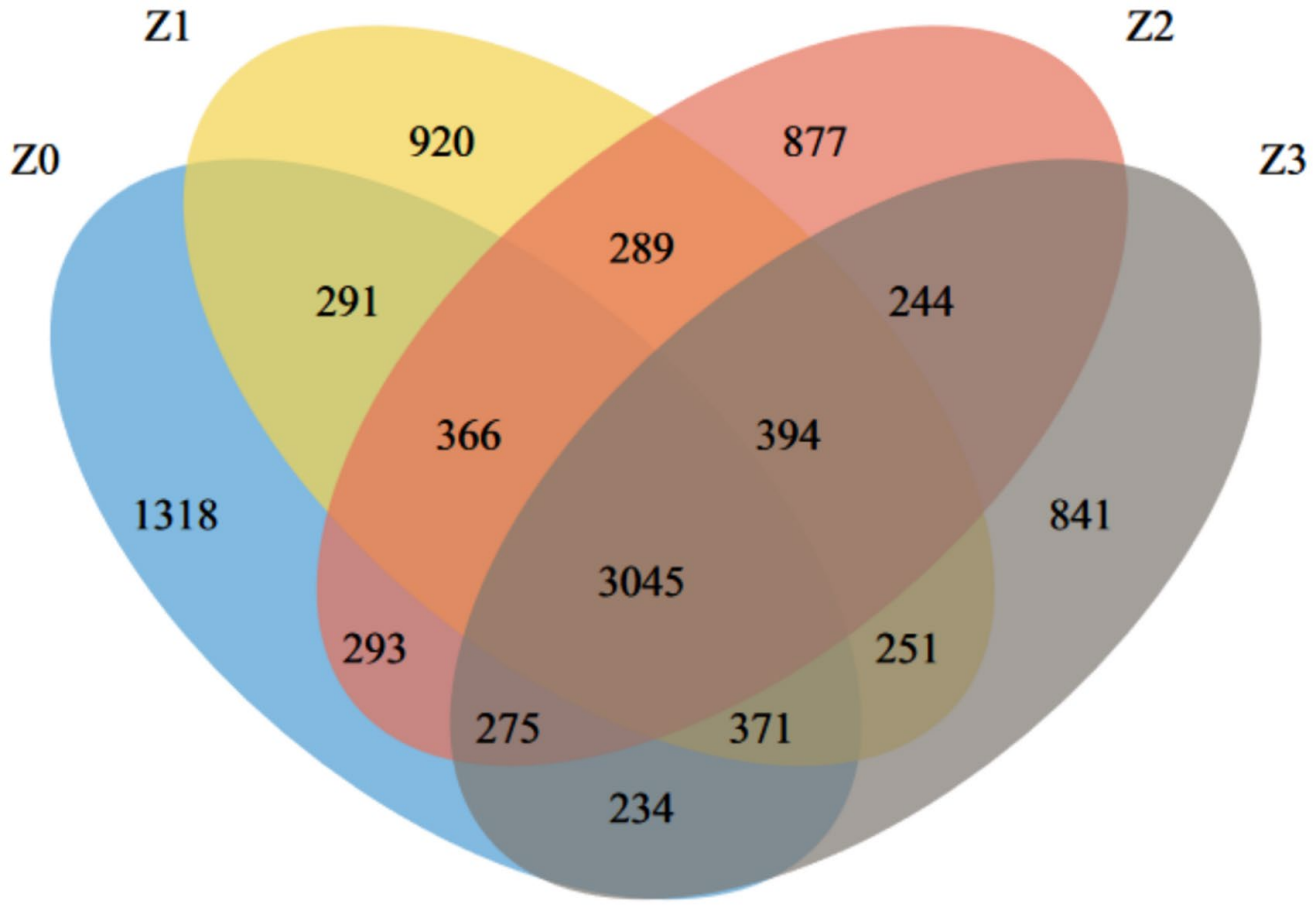
Distribution of OTU numbers across different treatments.

Rarefaction curve analysis showed that the curves for all samples leveled off (Supplementary Figure S1), indicating that the sequencing depth was sufficient to cover the community diversity and further supporting the reliability of the α-diversity and β-diversity analysis results in this study. A total of 10,009 OTUs were identified from the soil samples, with 3,045 core OTUs common to all four samples, representing 30.42% of the total OTUs. The distribution of bacterial OTUs in the samples was as follows: 1,318 in Z0, 920 in Z1, 877 in Z2, and 841 in Z3. The results revealed distinct microbial compositions among the habitats of normally cultivated *P. umbellatus* (Z0), the blank control (Z1), the *A. gallica-*only group (Z2) and the *P. umbellatus*-only group (Z3). Specifically, the number of OTUs in Z0 was significantly higher than that in Z1, Z2, and Z3 (Figure 1), suggesting increased bacterial community richness in the habitat of *P. umbellatus* and *A. gallica* during their symbiotic interaction.

**Figure 2:**
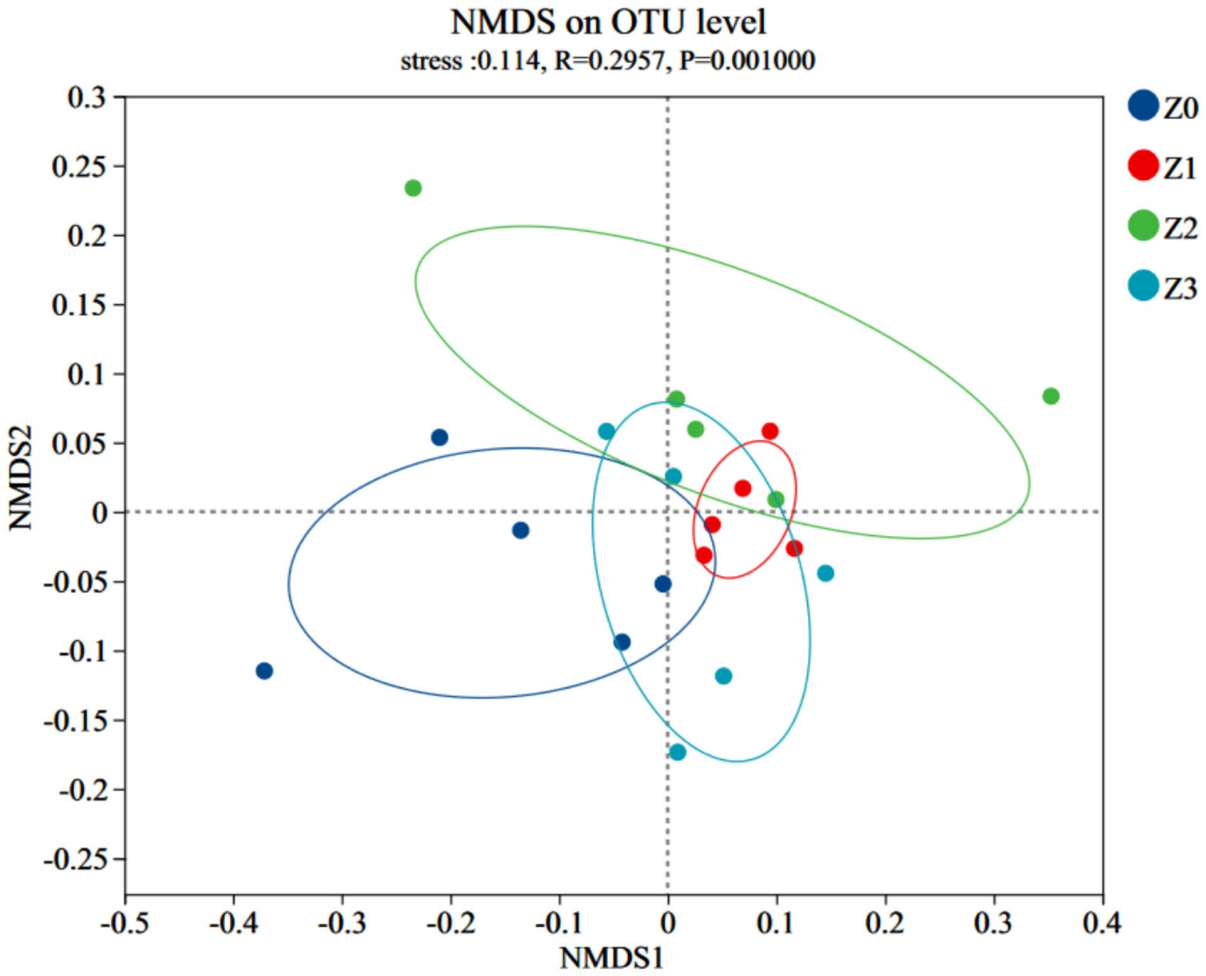
NMDS analysis of bacterial community structure.

**Figure 3:**
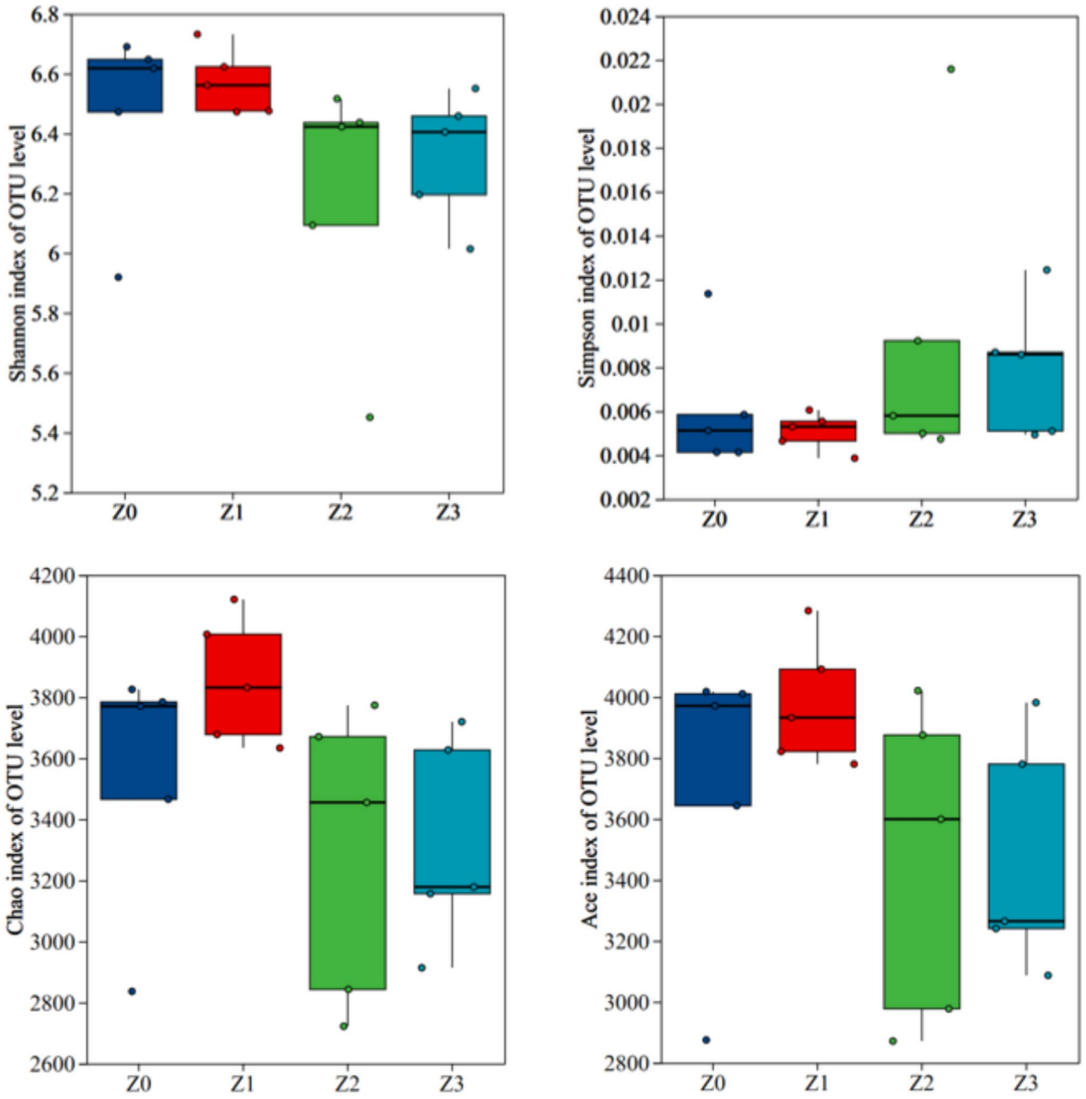
Alpha diversity indices of bacterial communities.

Furthermore, a non-metric multidimensional scaling (NMDS) analysis using the Bray-Curtis distance was conducted to illustrate consistent differences in β-diversity. The stress value for bacterial NMDS was 0.114, which is below the recommended threshold of 0.2 (Figure 2). The distinct clustering of bacterial communities observed in the four soil samples confirmed the impact of symbiosis between *P. umbellatus*and *Armillaria* on the bacterial communities during *P. umbellatus* growth. Figure 3 displays the alpha indices of the four soil samples, encompassing the richness estimator (Chao1 and ACE) and the diversity indices (Shannon and Simpson). The ACE index and Chao1 index of group Z0 exceeded those of groups Z2 and Z3, approximating the values of group Z1. The Shannon index of group Z0 surpasses that of groups Z2 and Z3, akin to group Z1. Conversely, the Simpson index of group Z0 is lower than those of groups Z2 and Z3, but comparable to that of group Z1. Despite these trends, the differences did not reach statistical significance (*p* > 0.05). These results corroborate the higher bacterial richness in group Z0 compared to groups Z2 and Z3, while being akin to group Z1.

**Figure 4:**
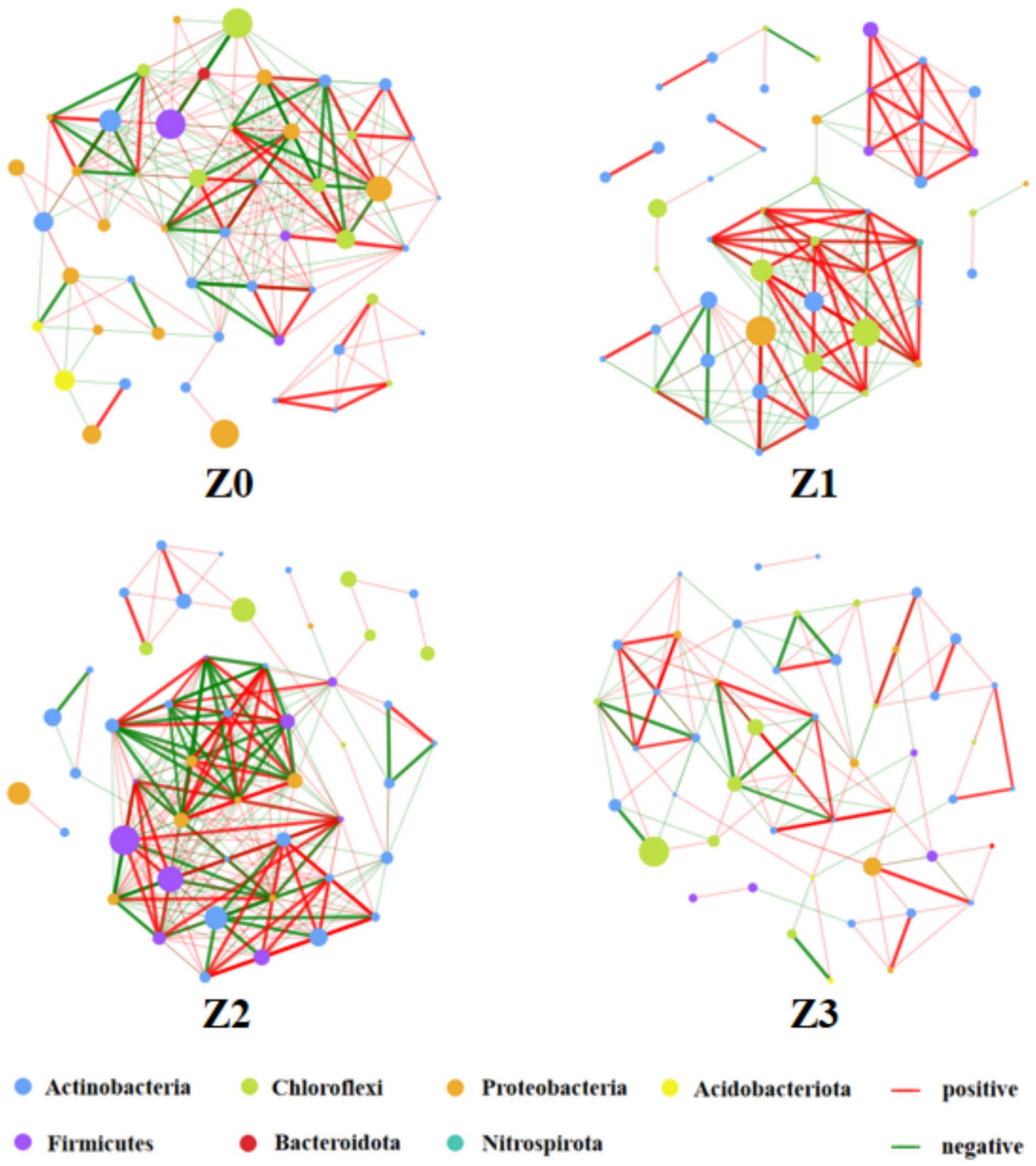
Co-occurring interactions of bacterial taxa.

The co-occurring bacterial interaction network indicates the influence of the symbiosis between *P. umbellatus* and *A. gallica* on the bacterial interactions in the vicinity of *P. umbellatus* sclerotia (Figure 4; Supplementary Table S1). The phyla Actinobacteria, Chloroflexi, Proteobacteria, Acidobacteriota, Firmicutes and Bacteroidota frequently interacted in Z0. Moreover, the relative abundance of Proteobacteria, Acidobacteriota, and Bacteroidota was higher in Z0 compared to Z1, Z2, and Z3 (Supplementary Table S2), indicating that the abundance and diversity of Proteobacteria, Acidobacteriota and Bacteroidota may be crucial for establishing a symbiotic relationship between *P. umbellatus* and *A. gallica*. The genus-level analysis revealed significant differences in bacterial community composition among the four groups. Specifically, 34, 37, 11, and 13 genera exhibited in groups Z0, Z1, Z2, and Z3, respectively (Supplementary Figure S2). Moreover, certain bacterial genera, such as *Mycobacterium*, unclassified_f__Xanthobacteraceae, *Bradyrhizobium*, *Flavobacterium*, *Acidothermus*, *Clostridium_sensu_stricto_1*, *Burkholderia-Caballeronia-Paraburkholderia*, *Devosia*, and unclassified_f__Blastocatellaceae, displayed significantly elevated relative abundances in the Z0 group compared to the other control groups, suggesting that these genera may contribute to the promotion of the symbiotic relationship establishment between *P. umbellatus* and *A. gallica* (Supplementary Table S3).

### Isolation and identification of rhizosphere soil bacteria and their effects on the growth and branching of *Armillaria gallica* rhizomorphs

**Figure 5:**
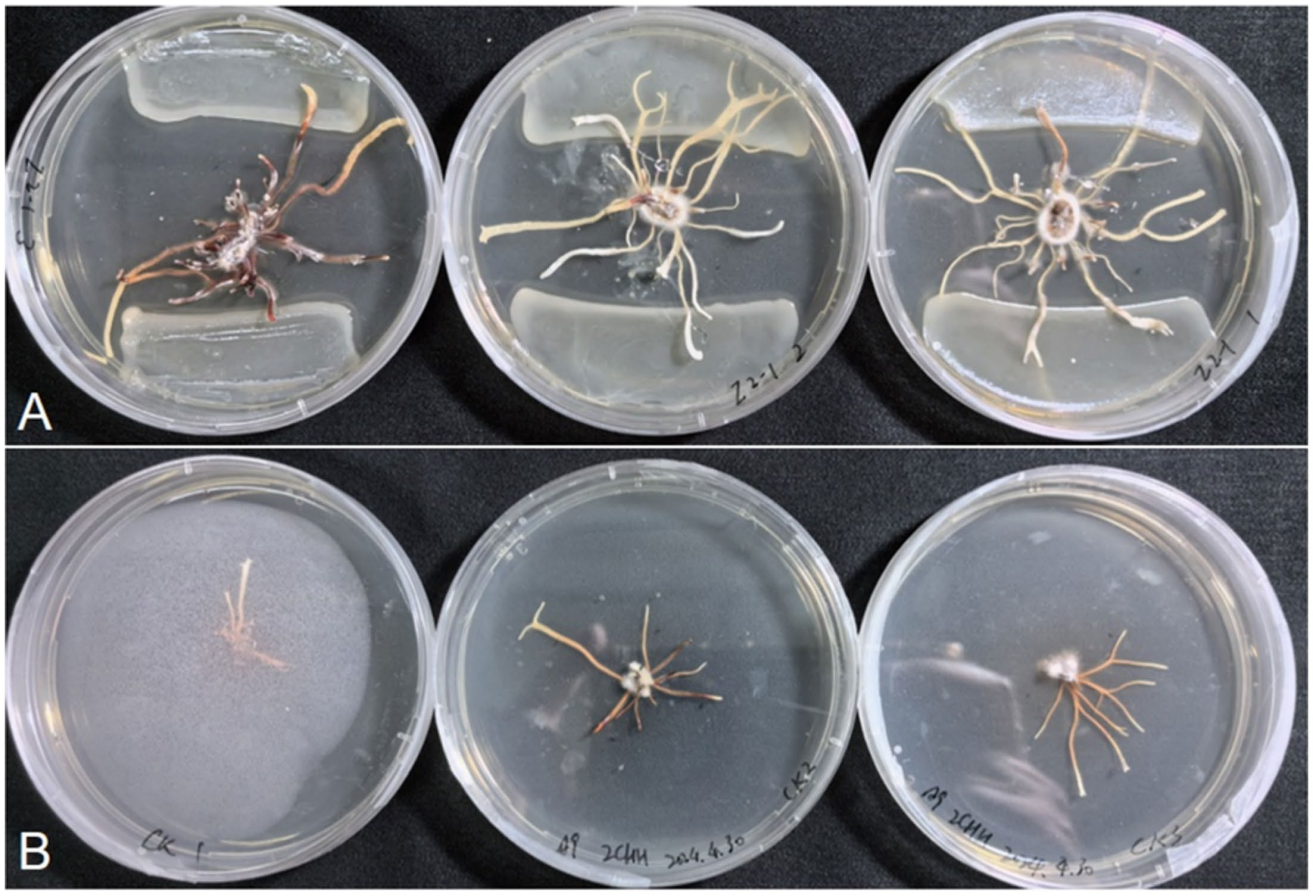
Growth-promoting effects of the strain Z2-1. **(A)** Co-culture of the strain Z2-1 and *A. gallica*. **(B)** CK: *A. gallica* rhizomorph cultured alone.

A total of 21 bacterial strains were isolated from the rhizosphere soil of *P. umbellatus*, followed by a co-culture experiment with *A. gallica* rhizomorphs and the bacterial isolates. The results are summarized in Supplementary Table S3. Among these bacterial isolates, 5 inhibited rhizomorph growth in *A. gallica*, while 11 reduced the number of rhizomorph branches. Notably, only one bacterial isolate (Z2-1) both promoted rhizomorph growth and increased the number of rhizomorph branches in *A. gallica* (Figure 5). The rhizomorph growth diameter in the culture dish was 48.18 mm, with 22.7 rhizomorph branches for isolate Z2-1, in contrast to the control group (CK) values of 22.84 mm and 8.7, respectively (Supplementary Table S4). Through EzBioCloud analysis, Z2-1 was identified as *Rhodococcus* sp. (Supplementary Table S5). Given the negligible recovery of fungal isolates in the preliminary isolation efforts, we propose that the growth of *A. gallica* is directly facilitated by bacterial associates.

### Z2-1 promotes the establishment of symbiotic relationship

The impact of Z2-1 inoculation on the establishment of symbiosis between *P. umbellatus* and *A. gallica* was assessed by examining the contact rate between *P. umbellatus* sclerotia and *A. gallica* rhizomorphs in pot experiments. The results demonstrated a significantly higher contact rate in the inoculated group compared to the control group (Supplementary Figure S3). Specifically, in the inoculated group, each container contained 7–9 sclerotia of *P. umbellatus*, all of which were in contact with *A. gallica* rhizomorphs, resulting in a contact rate of 100%. In contrast, the control group exhibited much lower contact rates, ranging from 0 to 22.2% (Table 1). These findings indicate that Z2-1 inoculation significantly improved the establishment of symbiosis between *P. umbellatus* and *A. gallica*. A chi-square test was performed to assess the significance of the differences in contact rates between the inoculated and control groups. The analysis indicated a highly significant disparity between the two groups (χ^2^ = 45.0, df = 1, *p* < 0.001), confirming that Z2-1 inoculation markedly promoted the establishment of symbiosis between *P. umbellatus* and *A. gallica*.

**Table 1:** Contact rates between *P. umbellatus* sclerotia and *A. gallica* rhizomorphs.

Group (treatment)	Container No.	Total sclerotia	Sclerotia in contact with rhizomorphs	Contact rate (%)
Inoculated (Z2-1)	1	8	8	100
	2	9	9	100
	3	7	7	100
	4	9	9	100
	5	7	7	100
Control	1	9	2	22.2
	2	8	0	0
	3	10	1	10
	4	6	0	0
	5	7	0	0

## Discussion

Our study offers significant insights into the intricate interplay between the medicinal fungus *P. umbellatus* and the fungus *A. gallica*, shedding light on the underlying dynamics of the soil microbial community. Specifically, our findings underscore the pivotal role of symbiosis in shaping the bacterial communities within the soil environment of *P. umbellatus*. The higher number of OTUs in the Z0 group, representing normally cultivated *P. umbellatus*, compared to the control groups (Z1, Z2, and Z3), suggests that the presence of both *P. umbellatus* and *A. gallica* establishes a distinct ecological niche that fosters a more diverse and intricate bacterial community. This observation is further supported by the NMDS analysis, which revealed distinct clustering of bacterial communities in the different soil samples, underscoring the substantial influence of symbiosis between *P. umbellatus* and *A. gallica* on the overall structure and composition of the soil microbiome. These findings align with our initial hypothesis positing that symbiosis plays a pivotal role in shaping the soil microbiome, as outlined in the introduction.

The α-diversity indices offer valuable insights into the abundance and diversity of bacterial communities across the experimental groups. Although the differences were not statistically significant (*p* > 0.05), potentially influenced by sample size or specific statistical methodologies employed, the elevated ACE, Chao1, and Shannon indices in the Z0 group compared to Z2 and Z3 suggest that the presence of *P. umbellatus* and *A. gallica* together promotes a higher level of bacterial richness and diversity. This finding aligns with previous studies demonstrating that symbiotic relationships can enhance microbial diversity by providing supplementary resources and habitats for microbial establishment and proliferation (Liu et al., 2021; Albornoz et al., 2022; Bender et al., 2019). The relatively higher abundance of certain bacterial phyla, such as Proteobacteria, Acidobacteriota, and Bacteroidota, in the Z0 group emphasizes their pivotal role in supporting the symbiotic relationship. These phyla are known for their functional capabilities, including nitrogen fixation, organic matter decomposition, and nutrient cycling, which could potentially contribute to the establishment and maintenance of the symbiosis between *P. umbellatus* and *A. gallica* (Li et al., 2025; Mukhia et al., 2024; Zhu et al., 2025). Subsequent investigations should encompass larger sample sizes and employ advanced statistical methodologies to validate and expand upon these results.

The co-occurring bacterial interaction network analysis revealed that certain bacterial genera, including *Mycobacterium*, *Bradyrhizobium*, and *Flavobacterium*, exhibited higher abundance in the Z0 group and likely play crucial roles in promoting the symbiotic relationship between *P. umbellatus* and *A. gallica*. These genera are known for their beneficial traits, such as the production of plant growth-promoting substances, enhancement of nutrient availability, and suppression of plant pathogens. Notably, *Bradyrhizobium* is well-known for its nitrogen-fixing capabilities, as they can serve as a vital nutrient source for the plant-fungus symbiotic system (Cheng et al., 2023). *Mycobacterium* and *Flavobacterium* are frequently associated with the degradation of complex organic compounds, potentially aiding in nutrient recycling within the soil ecosystem and bolstering the growth of both *P. umbellatus* and *A. gallica* (Terzaghi et al., 2021; González et al., 2011). Subsequent investigations should prioritize unraveling the precise mechanisms through which these bacterial genera enhance the symbiotic relationship and investigate their prospective utility in sustainable agricultural practices.

The isolation and identification of bacterial strains from the rhizosphere soil of *P. umbellatus* provided an opportunity to directly assess the impact of specific bacteria on the growth of *A. gallica* rhizomorphs. Notably, only one bacterial isolate, identified as *Rhodococcus* sp., significantly promoted both rhizomorph growth and branching (Ujvári et al., 2025). This finding underscores the potential for specific bacterial taxa to directly and positively influence the fungal symbiont. Given the generally low recovery rate of fungal isolates in the preliminary isolation efforts, this result is particularly intriguing and suggests that bacterial associates may play a predominant role in facilitating the growth and development of *A. gallica*. *Rhodococcus* is renowned for its diverse metabolic capabilities, which encompass the production of bioactive compounds and proficiency in degrading a broad spectrum of organic substrates (Cappelletti et al., 2020). These attributes have the potential to enhance nutrient availability and other growth-promoting factors for *A. gallica*, thereby stimulating its mycelial growth and branching. Future research should focus on elucidating the specific mechanisms by which *Rhodococcus* sp. promotes the growth of *A. gallica*, along with exploring the potential applications of this beneficial bacterium in sustainable agriculture. This may involve exploring the impact of bioactive compounds produced by *Rhodococcus* in nutrient cycling and fungal growth promotion, as well as assessing the broader ecological implications of introducing such beneficial bacteria into agricultural systems.

The significantly higher contact rate in the inoculated group suggests a pivotal role for Z2-1 in facilitating the interaction between *P. umbellatus* and *A. gallica*. This influence may stem from bioactive substances generated by Z2-1 or other mechanisms that enhance the growth and branching of *A. gallica* rhizomorphs, thereby increasing their contact with *P. umbellatus* sclerotia. These findings highlight the potential of leveraging beneficial bacteria such as Z2-1 to improve the efficiency of symbiotic establishment in agricultural and ecological systems. Subsequent investigations should concentrate on elucidating the specific mechanisms through which Z2-1 promotes symbiosis and exploring its practical applications in sustainable agriculture.

The results of our study carry significant ecological and agricultural implications. From an ecological perspective, the findings emphasize the interconnectedness of macrofungal, symbiotic fungal and bacterial communities within soil ecosystems. This interconnectedness underscores the necessity for a comprehensive comprehension of these relationships to grasp the dynamics of both natural and cultivated ecosystems fully. The symbiotic relationship between *P. umbellatus* and *A. gallica*, along with the associated bacterial communities, represents a complex network of interactions that contribute to the overall stability and resilience of the soil ecosystem. In an agricultural context, the identification of specific bacterial strains capable of stimulating the growth of *A. gallica* and, consequently, the vitality of *P. umbellatus*, holds substantial promise for sustainable agriculture. For example, these beneficial bacteria could be harnessed as biofertilizers or soil enhancements to boost plant growth and yield, diminish dependence on synthetic fertilizers, and foster more sustainable agricultural methodologies (Asif et al., 2024; Carril et al., 2025; Wang et al., 2022). Future research should focus on exploring the potential applications of these beneficial bacteria in agricultural systems, encompassing their utilization as biofertilizers and their contributions to soil health and plant development.

While our study offers valuable insights into the microbial communities associated with the *P. umbellatus*-*A. gallica* symbiosis, it is important to acknowledge several limitations. A notable limitation is the lack of statistical significance in the α-diversity indices (*p* > 0.05), potentially influenced by the relatively modest sample size or the specific statistical methodologies utilized (Wang et al., 2024). This suggests the importance of future research focusing on augmenting the sample size and employing more sophisticated statistical approaches to validate our findings. Additionally, the challenge of a generally low recovery rate of fungal isolates in the initial isolation endeavors is a common issue encountered in microbial ecology studies (Liu et al., 2024). This underscores the necessity for enhanced isolation techniques and experimental designs to enhance the recovery of fungal isolates. Furthermore, a more in-depth exploration of the functional mechanisms that underlie the observed impacts of specific bacterial genera and isolates on the symbiotic interactions between *P. umbellatus* and *A. gallica* is essential. This could involve detailed genomic and metabolic analyses to understand how these bacteria influence fungal growth and symbiotic establishment. The exploration of potential applications of these beneficial bacteria in agricultural systems could pave the way for novel approaches to sustainable crop production and soil management (Sun et al., 2023; Mahmud et al., 2021). Future research should incorporate field trials and long-term assessments to evaluate the practical feasibility and enduring effects of utilizing beneficial bacteria as biofertilizers or soil amendments.

## Conclusion

This study reveals the crucial role of specific bacteria in the symbiosis between *P. umbellatus* and *A. gallica*. The presence of both fungi significantly enriches rhizosphere bacterial communities, as evidenced by elevated diversity indices (ACE, Chao1, Shannon) in the symbiotic group (Z0). Co-occurrence analysis highlights the abundance of Proteobacteria, Acidobacteriota, and Bacteroidota in Z0. Co-culture experiments demonstrate the growth-promoting effects of *Rhodococcus* sp. Z2-1, an isolated strain, on *A. gallica* rhizomorph, leading to a substantial increase in diameter by 112.2% and branches number by 160.9%. Pot experiments validate the symbiotic enhancement by Z2-1, resulting in a 100% contact rate between *P. umbellatus* and *A. gallica*. These findings suggest practical applications in sustainable agriculture, including employing beneficial bacteria such as Z2-1 as biofertilizers to improve fungal cultivation. Future research should focus on elucidating the mechanisms of these effects and examining the broader agricultural applications.

## Data Availability

The data presented in the study are deposited in the SRA repository, accession number PRJNA1313114.
