# Supplementary material for: Symbiotic relationship between Polyporus umbellatus and Armillaria gallica shapes rhizosphere bacterial community structure and promotes fungal growth
Source: Front Microbiol. 2025 Sep 11;16:1658060. doi: 10.3389/fmicb.2025.1658060 (PMC12461091; doi:10.3389/fmicb.2025.1658060)
Supplement: Supplementary file 1 [file Data_Sheet_1.pdf]

Symbiotic relationship between *Polyporus umbellatus* and *Armillaria gallica* shapes rhizosphere bacterial community structure and promotes fungal growth

Lingfeng Zhou[1], Liu Liu[1], Weiwei Gao[1], Bing Li[1,2,*], Shunxing Guo[1,*]

[1]*Institute of Medicinal Plant Development, Chinese Academy of Medical Sciences and Peking Union Medical College, Beijing 100193, China*

[2]*State Key Laboratory for Quality Ensurance and Sustainable Use of Dao-di Herbs, Beijing, 100700, P. R. China*

*Correspondence:

E-mail address: zudengtianxia@126.com (B.L.); sxguo1986@163.com (S.G.)

Table S1. Correlation and topological properties of rhizosphere microbial communities of *P. umbellatus* under different cultivation methods.

Network properties	Z0	Z1	Z2	Z3
Number of nodes	48	46	49	50
Number of edges	199	181	242	123
Average degree	8.29	7.87	9.88	4.92
Graph density	0.18	0.17	0.21	0.10
Average clustering coefficient	0.54	0.65	0.71	0.60

Table S2. Top 10 phylum in terms of relative abundance of bacteria (%).

Taxonomy	Z0(%)	Z1(%)	Z2(%)	Z3(%)
Actinobacteriota	27.4	34.5	34.7	33.6
Chloroflexi	17.1	23.4	17.7	25.7
Proteobacteria	25.4	17.1	19.0	15.7
Firmicutes	6.63	6.51	15.5	7.39
Acidobacteriota	9.94	5.62	3.15	7.08
Patescibacteria	3.71	3.09	2.59	2.32
Bacteroidota	3.58	1.81	2.47	1.94
Myxococcota	1.44	1.90	1.36	1.30
Gemmatimonadota	0.80	1.32	0.64	0.69
Verrucomicrobiota	1.03	0.82	0.51	0.89
others	2.98	3.94	2.41	3.38

Table S3. Top 30 genera in terms of relative abundance of bacteria (%).

Taxonomy	Z0(%)	Z1(%)	Z2(%)	Z3(%)
unclassified_c__KD4-96	4.90	8.10	5.55	12.8
unclassified_o__Gaiellales	4.16	7.51	6.98	5.27
unclassified_f__Roseiflexaceae	3.44	5.90	3.51	3.92
Bacillus	1.88	3.02	8.37	3.49
Sphingomonas	3.23	4.28	3.95	3.86
Gaiella	1.52	4.43	3.40	3.30
unclassified_f__JG30-KF-CM45	1.49	1.93	2.05	2.53
unclassified_f__67-14	1.81	1.94	2.07	2.09
unclassified_o__Vicinamibacterales	2.77	1.65	0.83	2.08
unclassified_f__A4b	2.10	1.44	1.90	1.31
Paenibacillus	0.99	1.12	2.46	1.17
unclassified_c__TK10	1.43	1.46	1.29	1.29
Mycobacterium	2.09	1.00	1.16	1.21
Allorhizobium-Neorhizobium-Pararhizobium-Rhizobium	2.01	0.52	2.07	0.69
Conexibacter	1.29	1.43	1.40	1.13
Pseudonocardia	1.11	1.39	1.26	1.25
Unclassified_f__Micromonosporaceae`	0.92	0.89	1.82	1.31
Solirubrobacter	0.67	1.33	1.28	1.44
unclassified_o__Saccharimonadales	1.34	1.29	1.17	0.89
unclassified_f__Xanthobacteraceae	1.98	0.87	0.64	1.17
Galbitalea	1.25	0.30	1.87	1.13
Nocardioides	0.91	1.11	1.02	1.47
Blastococcus	0.50	1.48	0.98	0.96
Bradyrhizobium	1.59	0.54	0.66	0.52
Flavobacterium	1.16	0.41	0.91	0.69
Acidothermus	1.45	0.48	0.46	0.49

Clostridium_sensu_stricto_1	2.06	0.21	0.30	0.20
Burkholderia-Caballeronia-Paraburkholderia	1.19	0.25	0.45	0.28
Devosia	1.21	0.09	0.37	0.21
unclassified_f__Blastocatellaceae	1.29	0.21	0.16	0.19
Sporosarcina	0.12	0.28	1.16	0.16
Others	46.1	43.1	38.5	41.6

Table S4. The impact of bacteria on the growth of *A. gallica*

Isolates	Rhizomorph branching number	Diameter of rhizomorph growth (mm)
CK	8.7±1.5	22.84±8.16
D0-1	5.3±4.0	20.96±14.22
D0-10	2.7±2.5*	8.49±4.19*
D0-3	4.0±4.6	14.00±11.62
D0-4	4.7±4.5	17.62±7.93
D1-10	1.0±1.7**	11.27±6.42
D1-18	1.7±2.1*	10.34±5.32
D1-2	6.0±5.3	12.23±5.26
D1-9	2.7±2.3*	10.80±3.49
G0-3	11.3±3.2	16.40± 5.36
G0-7	3.0±3.6*	7.41±3.26*
G0-8	0.7±1.2**	6.05±3.05*
G1-18	6.3±3.2	34.23±8.92
G2-14	0.0±0.0**	0.00±0.00***
G2-2	6.7±6.1	16.73±16.67
G3-10	2.0±2.0*	13.51± 3.79
G3-12	2.3±1.2*	11.11±3.63
G3-3	4.3±4.5	16.38±10.54
G3-8	7.7±2.5	31.99±10.91
Z0-5	1.3±2.3**	10.36±4.56
Z0-6	0.3±0.6**	8.16±3.92*
Z2-1	22.7±3.1***	48.18±5.00***

Compared with the CK group, *, $P < 0.05$; **, $P < 0.01$; ***, $P < 0.001$

Table S5. Identification of Z2-1 using EzBioCloud.

Isolate	Top-hit taxon	Similarity (%)	Completeness (%)
Z2-1	*Rhodococcus pedocola*	99.14	100.0

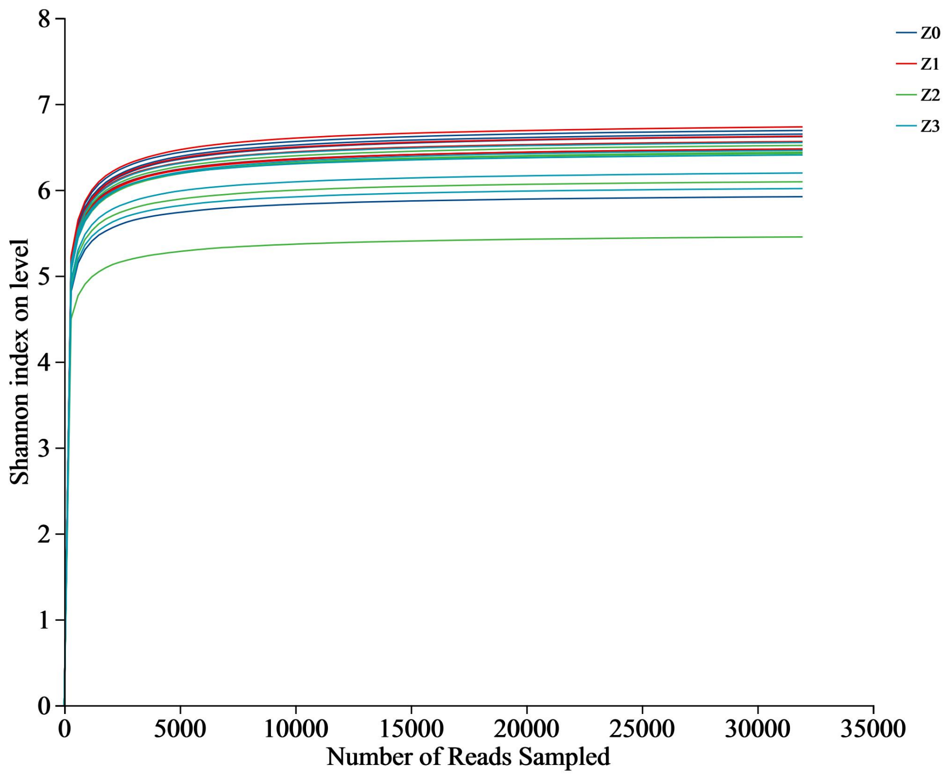

Fig. S1 Rarefaction curves demonstrating sufficient sequencing depth for community diversity coverage.

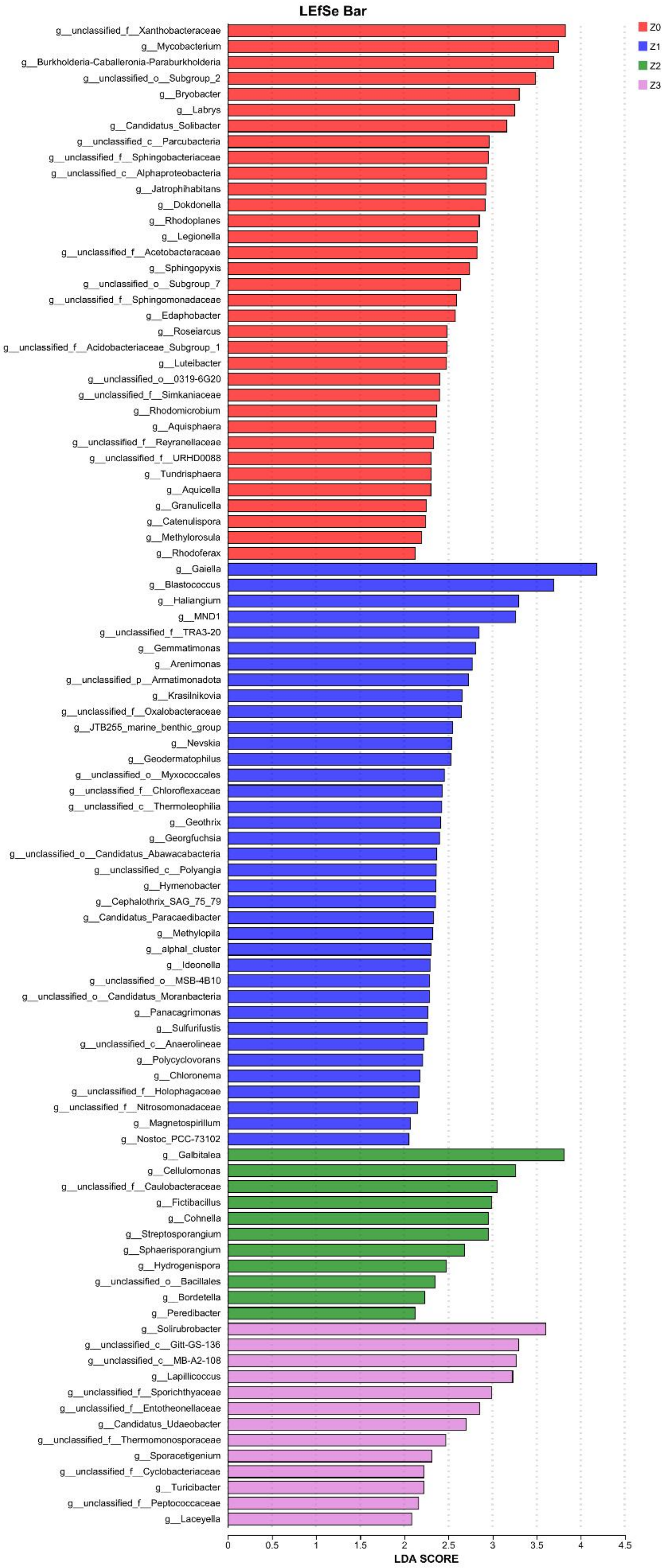

Fig. S2 LDA score distribution histogram. The histogram illustrates the LDA scores of significantly different taxa identified by LEfSe analysis. The length of each bar represents the LDA score, indicating the effect size of the taxon.

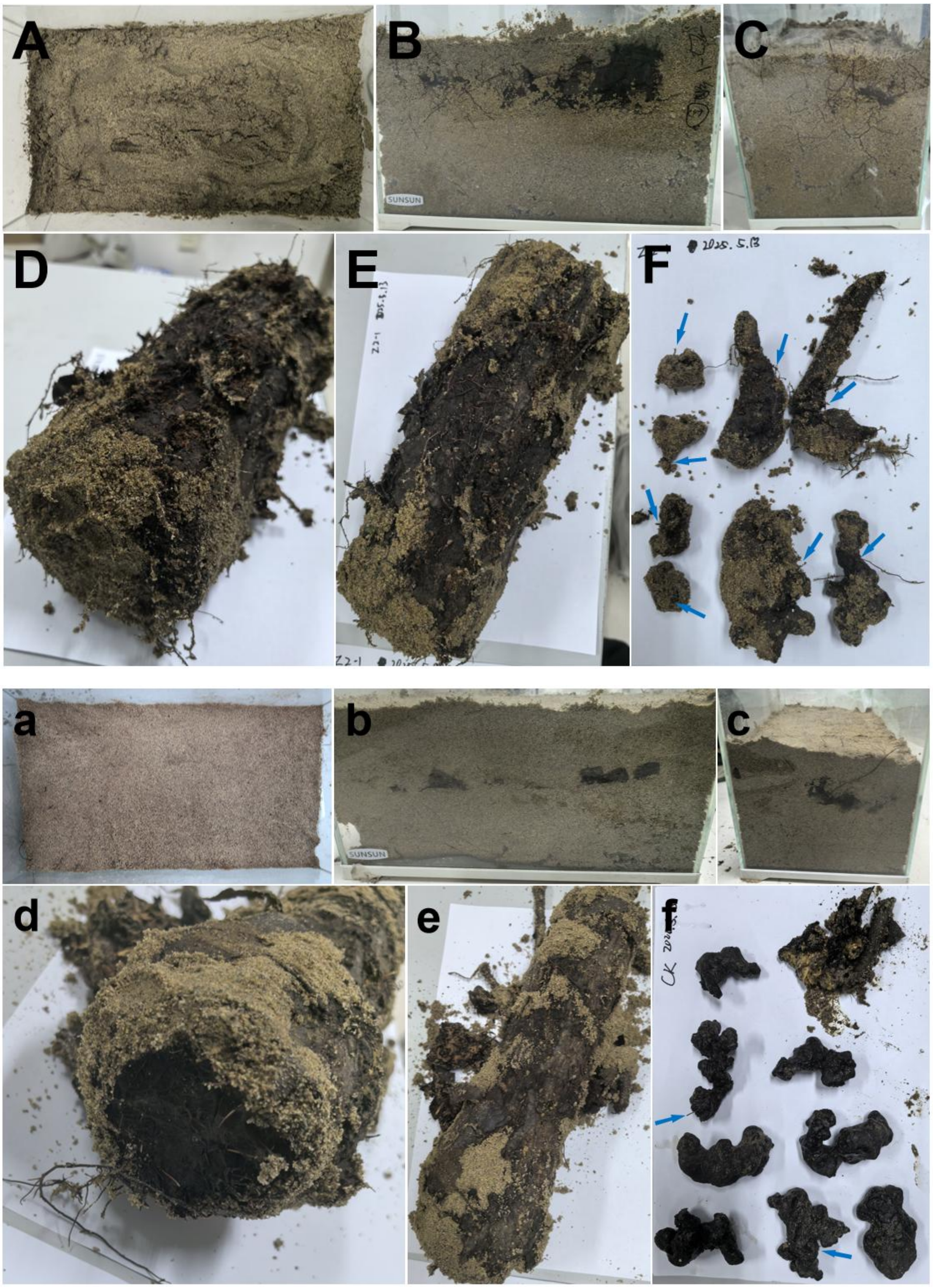

Fig. S3 Effect of strain Z2-1 on the establishment of symbiosis between *P. umbellatus* and *A. gallica*. Capital letters denote experimental group images, while lowercase letters denote control group images. Panels Aa, Bb, and Cc show the three-view diagrams of *P. umbellatus* planted in glass containers. In the experimental group containers, a large number of rhizomorphs can be observed at the edges, while

rhizomorphs were rarely seen in the control group containers. Panels Dd and Ee illustrate the colonization degree of *A. gallica* on wooden sticks. In the experimental group, the wooden sticks are heavily colonized by rhizomorphs, while rhizomorph colonization was rarely observed in the control group. Panels Ff display the symbiotic status between *P. umbellatus* and *A. gallica*. In the experimental group, all *P. umbellatus* and *A. gallica* rhizomorphs were in contact, while such contact between *P. umbellatus* and *A. gallica* rhizomorphs is rarely observed in the control group. The contact points between *P. umbellatus* and *A. gallica* were indicated by blue arrows.
